# Male mice emit distinct ultrasonic vocalizations when the female leaves the social interaction arena

**DOI:** 10.3389/fnbeh.2013.00159

**Published:** 2013-11-19

**Authors:** Mu Yang, Darren Loureiro, David Kalikhman, Jacqueline N. Crawley

**Affiliations:** ^1^Department of Psychiatry and Behavioral Sciences, University of California Davis School of MedicineCalifornia, CA, USA; ^2^Laboratory of Behavioral Neuroscience, National Institute of Mental HealthBethesda, MD, USA

**Keywords:** ultrasonic vocalizations, USV, mouse models of autism, mouse model of communication, social interaction, social behaviors

## Abstract

Adult male mice emit large number of complex ultrasonic vocalizations (USVs) when interacting with adult females. Call numbers and call categories differ greatly among inbred mouse strains. Little is known about USV emissions when the social partner departs. To investigate whether call repertoires and call rates are different when the male is interacting with a female and after the removal of the female, we designed a novel male-female social interaction test in which vocalizations were recorded across three phases. During phase 1, the male subject freely interacts with an unfamiliar estrus female mouse in a clean cage for 5 min. During phase 2, the female is removed while the male remains in the cage for 3 min. During phase 3, the same female is returned to the cage to rejoin the male subject mouse for 3 min. C57BL/6J (B6), FVB.129P2-Pde6b(+) Tyr(c-ch)/Ant (FVB), and BTBR T+ tf/J (BTBR) male subject mice were tested in this paradigm. All three strains emitted USVs during their initial interaction with the female partner. When the female was reintroduced in phase 3, numbers of USVs were similar to the initial introductory phase 1. Strain comparisons indicated fewer calls in pairs of BTBR males and stimulus females than in pairs of B6 males and stimulus females and pairs of FVB males and stimulus females. In the absence of the female, all FVB males vocalized, while only one third of B6 males and one third of BTBR males vocalized. In all three strains, changes in call category repertoires were detected after the female was removed. Call categories reverted to the phase 1 pattern when the female was returned in phase 3. Present findings indicate that males of commonly used inbred strains emit USVs when a partner female leaves the testing arena, suggesting that removing a salient social stimulus may be a unique approach to elicit USVs from mice. Our three-phase paradigm may also be useful for studying attention to social cues, and qualitative differences in vocalizations when a social partner is present vs. suddenly absent.

## Introduction

Mice emit ultrasonic vocalizations (USVs) throughout lifetime. Pups emit USVs when separated from the nest, functionally to elicit retrieval and nursing behaviors from the dam (Noirot, 1972; D'Amato et al., 2005; Ehret, 2005; Thornton et al., 2005; Scattoni et al., 2008; Young et al., 2010; Okabe et al., 2013). Juvenile and adult mice emit USVs during same-sex social interactions (Maggio and Whitney, 1985; D'Amato and Moles, 2001; Moles et al., 2007; Panksepp et al., 2007; Scattoni et al., 2011; Hammerschmidt et al., 2012a; Ey et al., 2013) and male-female social interactions (Holy and Guo, 2005; Kikusui et al., 2011; Scattoni et al., 2011; Sugimotom et al., 2011; Brielmaier et al., 2012; Ey et al., 2012; Hammerschmidt et al., 2012a; Hanson and Hurley, 2012; Yang et al., 2012b; Mahrt et al., 2013). The male-female interaction test is widely used to study vocal phenotypes in male mice. Considerable evidence indicates that USVs detected during this interaction are emitted by the male rather than the female (Whitney et al., 1973; White et al., 1998; Wang et al., 2008; Sugimotom et al., 2011). Nevertheless, until it is technically possible to distinguish calls emitted by different animals in the same arena, it cannot be ruled out that females do emit small number of calls when interacting with males. Besides live females, fresh female urinary odors and body odors are similarly effective in eliciting USVs from adult male mice (Nyby et al., 1977; Whitney and Nyby, 1979; Byatt and Nyby, 1986; Hoffmann et al., 2009; Malkesman et al., 2010; Roullet et al., 2011; Wohr et al., 2011).

Inbred mouse strains with high and low sociabilities differ greatly in USV call numbers and call categories as pups (Scattoni et al., 2008), during juvenile same-sex and mixed-sex social interactions (Panksepp et al., 2007), and during adult same-sex and male-female social interactions (Kikusui et al., 2011; Scattoni et al., 2011; Sugimotom et al., 2011; Scattoni et al., 2013). These findings suggest that vocalizations might be an important readout in studies of mouse social behaviors. Non-social stimuli such as environmental novelty, restraint stress, and painful stimuli were also effective in eliciting USVs (Kurejova et al., 2010; Chabout et al., 2012). The question of whether mouse USVs serve communicative functions is the focus of many recent studies (Arriaga et al., 2012; Hammerschmidt et al., 2012a,b; Arriaga and Jarvis, 2013; Mahrt et al., 2013). Unlike in rats, USVs in mice are not associated with aversive or positive states. It was suggested that USVs in mice are likely to facilitate or inhibit social interactions (Portfors, 2007).

Previous studies indicate that female mice exhibit more approach behaviors to male USVs than to pup USVs, artificial control sounds, or silence (Hammerschmidt et al., 2009; Shepard and Liu, 2011), and prefer vocalizing males over devocalized males (Pomerantz et al., 1983), suggesting that male USVs may have a role in facilitating courtship. Key questions about functions of USVs remain to be investigated. Which components of the male USV repertoire function to attract females? Do males reliably alter their call patterns in response to changes in the social context? Do different strains of males use different call components to attract females? To begin to address these questions about mouse USVs, we designed a novel three-phase male-female social interaction test. Phase 1 is a 5-min session, when the male subject freely interacts with an unfamiliar B6 estrous female mouse. Phase 2 is a 3-min session, when the male remains in the cage but the female is removed. Phase 3 is a 3-min session, when the same female is placed back into the cage to rejoin the male subject mouse. B6, FVB, and BTBR males were tested in the present study. These strains were selected for their distinct social behaviors. Numerous studies have reported high social behaviors in B6 and low social behaviors in BTBR (Yang et al., 2007, 2012a; McFarlane et al., 2008; Moy et al., 2008; Silverman et al., 2010a,b, 2013; Scattoni et al., 2011; Babineau et al., 2013). B6 and BTBR also differ greatly in USV parameters during social interactions. Compared to adult B6, adult BTBR emitted fewer USVs in same-sex and male-female social interactions. Further, call category repertoires were significantly different between the two strains in these social encounters (Scattoni et al., 2011, 2013). FVB.129P2-Pde6b(+) Tyr(c-ch)/Ant is a novel sighted FVB strain (Errijgers et al., 2007) that exhibits high social approach behaviors and high reciprocal social interaction behaviors (Silverman et al., 2010a), suggesting that this strain might be used as a corroborative high social control strain. While USVs have been studied in the original blind FVB/NJ substrain (Scattoni et al., 2008), USVs in the FVB/Ant substrain have not yet been reported.

## Materials and methods

### Subjects

All procedures were approved by the National Institute of Mental Health Animal Care and Use Committees. B6, FVB, and BTBR breeding pairs were purchased from the Jackson Laboratory (Bar Harbor, ME) and bred at NIMH in Bethesda, Maryland. Mice were weaned at 21 days of age, and group housed by sex in cages of 2–4 littermates per cage. Standard rodent chow and tap water were available *ad libitum*. In addition to standard bedding, a Nestlet square and a cardboard tube were provided in each cage. The colony room was maintained on a 12:12 light/dark cycle with lights on at 7:00 AM, and at ~20°C and 55% humidity. All experiments were conducted between 9:00 AM and 4:00 PM.

### The three-phase male-female social interaction test

One three-phase male-female social interaction test was developed based on the standard single phase male-female social interaction test (Kikusui et al., 2011; Scattoni et al., 2011; Ey et al., 2012; Yang et al., 2012b). All male subject mice and female stimulus mice were group-housed and sexually naïve at the time of testing. All animals were between 2 and 4 months of age at the time of testing. Females were visually inspected for estrous cycle, as previously described (Champlin et al., 1973; Caligioni, 2009; Scattoni et al., 2011; Brielmaier et al., 2012). Only those that were considered to be in proestrus or estrus (vagina is open and the tissue around is reddish-pink or pink) were used as stimulus mice. The test was conducted in a sound-attenuating environmental chamber (ENV-018V; Med Associates, St. Albans, VT, USA). Interior walls were covered with convoluted foam sheets (Uline, Pleasant Prairie, WI). Behaviors were recorded by a digital closed-circuit television camera (Panasonic, Secaucus, NJ, USA) positioned 30 cm horizontally from the cage. Dim red light illumination (10 lux) was used to provide illumination. USVs were recorded by an ultrasonic microphone (Avisoft UltraSoundGate condenser microphone capsule CM15; Avisoft Bioacoustics, Berlin, Germany) mounted 2 cm above the testing cage. Sampling frequency for the microphone was 250 kHz, and the resolution was 16 bits. Phase 1 is a 5-min free interaction session. The male subject mouse was removed from its home cage and placed in a clean standard mouse cage (14′ × 5.5′ × 4.5′) whose floor was covered with a thin layer of clean bedding. The testing cage was then placed in the sound-attenuating chamber without a cage lid. An estrus B6 female was introduced into the testing cage and the chamber door was closed immediately. At the end of phase 1, the B6 female was removed from the testing cage and placed in a clean cage outside the experiment room. The male subject was left alone in the testing cage for 3 min (phase 2). USVs emitted by the male in the absence of the B6 female were recorded. At the end of phase 2, the female was returned to the testing cage to rejoin the male subject for a 3-min free interaction session (phase 3). Each female stimulus mouse interacted with no more than two male subjects a day, with at least half an hour of resting between tests. Animals that were not being tested were kept out of the experimental room, in cages covered with lids. The door of the experimental room was tightly closed when USVs were recorded. Durations and frequencies of social behaviors were scored from videotapes, by a highly trained investigator using the Noldus Observer software (Noldus Information Technology, Leesburg, VA, USA). Behaviors analyzed were nose-to-nose sniffing (sniffing or snout contact in the region around snout/head/heck/mouth), anogenital sniffing (sniffing the anogenital area), body sniffing (sniffing the trunk or limbs), follow (walking at the same speed behind the other animal, keeping a distance of 2 cm or shorter). Bouts of arena exploration were scored a measure for novelty exploration.

### Analysis of ultrasonic vocalizations

USVs were analyzed using Avisoft SASLab Pro software (Avisoft Bioacoustics). Spectrograms were generated for each 1-min audio file, with a FFT-length of 512 points and a time window overlap of 75% (100% Frame, Hamming window). The spectrogram was generated at a frequency resolution of 488 Hz and a time resolution of 1 ms. A lower cut-off frequency of 15 kHz was used to reduce background noise outside the relevant frequency band to 0 dB. Calls were inspected visually by three investigators and classified into eight categories, generally based on criteria described previously (Scattoni et al., 2011). Inter-rater reliability was >95%. Categories analyzed in this study are: complex, two-component, upward, downward, chevron, short, frequency steps, and flat. “Unstructured” and “Composite” described in a previous study (Scattoni et al., 2011) were rarely detected in our study, and were not analyzed. Summary statistics were generated by Avisoft SASLab Pro and analyzed using STATISTICA (StatSoft, Inc.). For phase 1, all calls emitted by callers during minutes 1, 3, and 5 (7412 calls from 12 B6 callers, 7400 calls from 12 FVB callers, and 5702 calls from 9 BTBR callers) were categorized. For phase 2, all calls emitted by callers during the 3-min session (1857 calls from 4 B6 callers, 2094 calls from 12 FVB callers, and 1153 calls from 4 BTBR callers) were categorized. For phase 3, all calls emitted by callers during the 3-min session (4646 calls from 10 B6 callers, 4015 calls from 10 FVB callers, and 2608 calls from 9 BTBR callers) were categorized. For call number analysis, all audio files were included. Phase effects on number of calls within each strain were analyzed by comparing number of calls per minute across the three phases. Strain differences in number of calls were analyzed by comparing total calls in the first 3 min of phase 1, total calls in the 3 min of phase 2, and total calls in the 3 min of phase 3 among the three strains. For call category analysis, only audio files that contained detectable USVs were included. Similar approaches were described previously (Scattoni et al., 2011). Phase effects on call categories within each strain were analyzed by comparing average numbers and percentages of call each category across the three phases. Strain differences in call categories were analyzed by comparing total calls of each category in minutes 1, 3, and 5 of phase 1, total calls of each category in the 3 min of phase 2, and total calls of each category in the 3 min of phase 3 among the three strains.

### Statistical analysis

Repeated Measures ANOVA was used to compare social behaviors in phase 1 and 3 for each strain. USV data were not normally distributed in many cases. Non-parametric Kruskal-Wallis test was used to analyze data with non-normal distribution, to compare USV parameters in phases 1, 2, and 3 within each strain. One-Way ANOVA was used to analyze strain differences in behaviors in phases 1 and 3. Significant ANOVA results were followed by Student's Newman-Keuls test for *post-hoc* analysis, with Bonferroni correction for multiple comparisons. Kruskal-Wallis test was used to compare strain differences in USV data with non-normal distribution.

## Results

### USVs and behaviors in the presence and absence of the female

**Figure 1:**
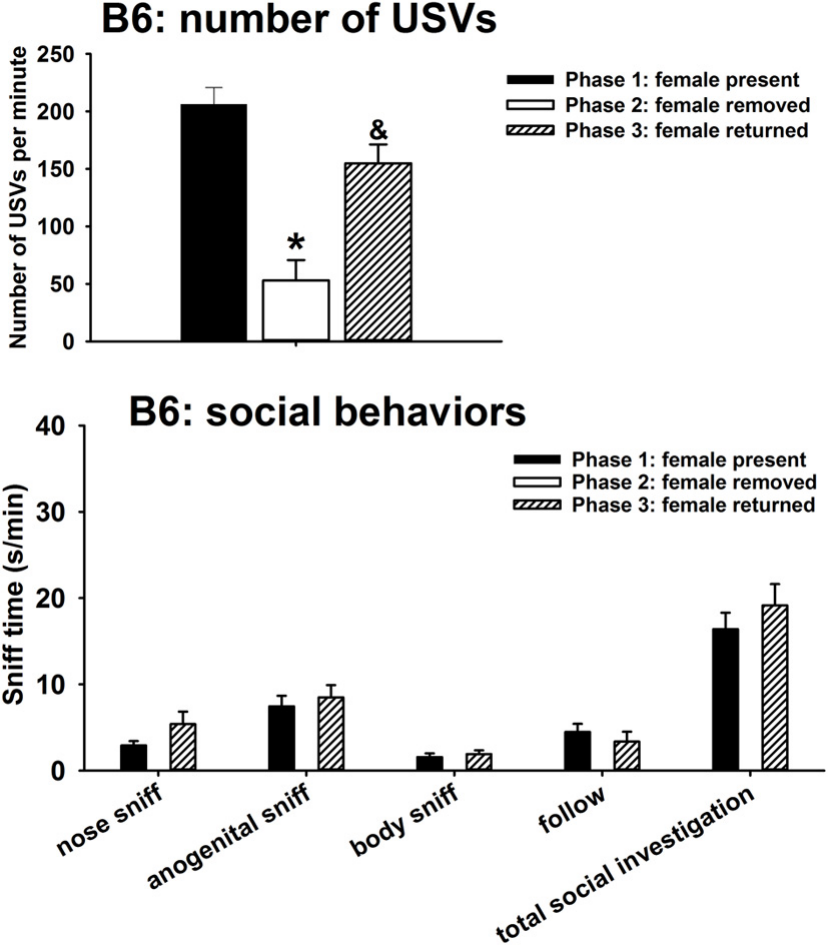
**USVs and social behaviors in B6 males during the first interaction with the female (phase 1), absence of the female (phase 2), and second interaction with the female (phase 3)**. USVs were detected from 12 out of 12 B6 in phase 1, 4 out of 12 B6 in phase 2, and 10 out of 10 B6 in phase 3. All animals were included in call number analysis and behavioral comparisons. B6 emitted fewer USVs in phase 2 than in phase 1, and more in phase 3 than in phase 2. Analysis of behavioral parameters revealed no significant differences between phase 1 and phase 3, on durations of nose-to-nose sniffing, anogenital sniffing, body sniffing, follow, and total social investigation. ^*^p < 0.05 vs. phase 1; ^&^*p* < 0.05 vs. phase 2.

A major result, that was not originally predicted, was the emission of calls by males of all three strains after the female was removed from the interaction arena. Figure 1 illustrates USVs and social behaviors in B6 males during the first interaction with the female (phase 1), absence of the female (phase 2), and second interaction with the female (phase 3). USVs were detected in 12 out of 12 B6 in phase 1, 4 out of 12 B6 in phase 2, and 10 out of 10 B6 in phase 3. Significant phase effects were found on number of USVs [*H*_(2)_ = 37.35, *p* < 0.001], with USVs per minute being significantly lower in phase 2 than in phase 1 (*p* < 0.001), and higher in phase 3 than in phase 2 (*p* < 0.001). USV numbers in phase 1 and phase 3 were not significantly different. Analysis of behavioral parameters revealed no significant differences between phase 1 and phase 3, on durations of nose-to-nose sniffing [*F*_(1, 11)_ = 3.69, NS], anogenital sniffing [*F*_(1, 11)_ = 0.22, NS], body sniffing [*F*_(1, 11)_ = 0.42, NS], follow [*F*_(1, 11)_ = 1.05, NS], and total social investigation [*F*_(1, 11)_ = 0.72, NS].

**Figure 2:**
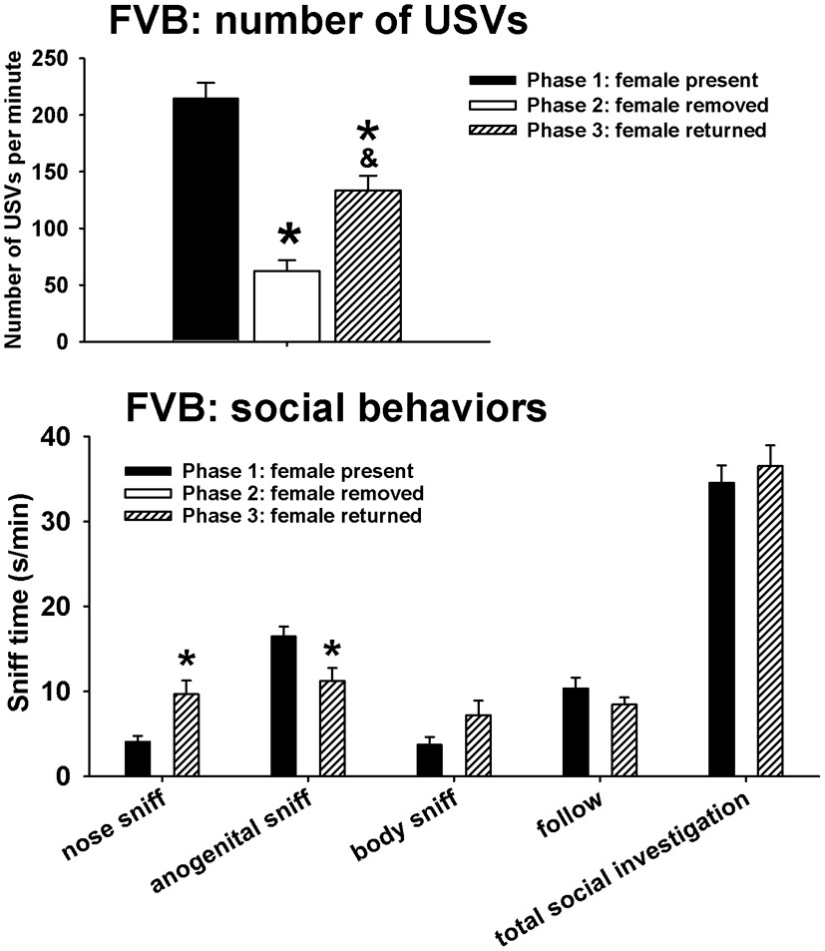
**USVs and social behaviors in FVB males in phases 1, 2, and 3**. USVs were detected from 12 out of 12 FVB in phase 1, 12 out of 12 FVB in phase 2, and 10 out of 10 FVB in phase 3. All animals were included in call number analysis and behavioral comparisons. FVB emitted fewer USVs in phase 2 and phase 3 than in phase 1, and more in phase 3 than in phase 2. Analysis of behavioral parameters revealed no significant differences between phase 1 and phase 3, on durations of body sniffing, follow, and total social investigation. FVB displayed higher levels of nose-to-nose sniffing and lower levels of anogenital sniffing in phase 3 than in phase 1. ^*^p < 0.05 vs. phase 1; ^&^*p* < 0.05 vs. phase 2.

Figure 2 illustrates USVs and social behaviors in FVB males during phases 1, 2, and 3. USVs were detected in 12 out of 12 FVB in phase 1, 12 out of 12 FVB in phase 2, and 10 out of 10 FVB in phase 3. Significant phase effects were found on number of USVs [*F*_(2, 98)_ = 42.41, *p* < 0.001], with USVs per minute being lower in phase 2 and phase 3 than in phase 1 (*p* < 0.001 for each comparison), and higher in phase 3 than in phase 2 (*p* < 0.01). Analysis of behavioral parameters revealed no significant phase effects on durations of body sniffing [*F*_(1, 9)_ = 1.82, NS], follow [*F*_(1, 9)_ = 0.52, NS], and total social investigation [*F*_(1, 9)_ = 0.65, NS]. FVB displayed higher levels of nose-to-nose sniffing [*F*_(1, 9)_ = 7.50, *p* < 0.05] and lower levels of anogenital sniffing in phase 3 than in phase 1 [*F*_(1, 9)_ = 8.02, *p* < 0.05].

**Figure 3:**
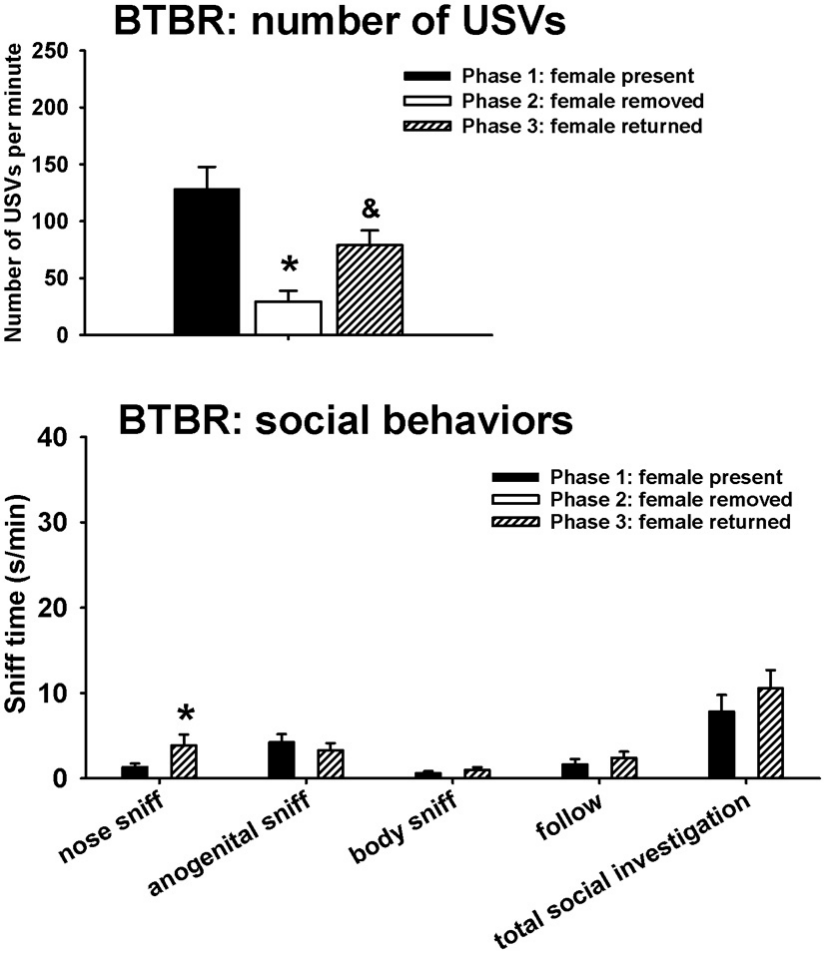
**USVs and social behaviors in BTBR males in phases 1, 2, and 3**. USVs were detected from 9 out of 11 BTBR in phase 1, 4 out of 11 BTBR in phase 2, and 9 out of 11 BTBR in phase 3. All animals were included in call number analysis and behavioral comparisons. BTBR emitted fewer USVs in phase 2 than in phase 1, and more in phase 3 than in phase 2. Analysis of behavioral parameters revealed no significant differences between phase 1 and phase 3, on durations of anogenital sniffing, body sniffing, follow, and total social investigation. BTBR displayed higher levels of nose-to-nose sniffing in phase 3 than in phase 1. ^*^p < 0.05 vs. phase 1; ^&^*p* < 0.05 vs. phase 2.

Figure 3 illustrates USVs and social behaviors in BTBR males during phases 1, 2, and 3. USVs were detected in 9 out of 11 BTBR in phase 1, 4 out of 11 BTBR in phase 2, and 9 out of 11 BTBR in phase 3. Significant phase effects were found on number of USVs [*H*_(2)_ = 21.89, *p* < 0.001], with USVs per minute being lower in phase 2 than in phase 1 (*p* < 0.001), and higher in phase 3 than in phase 2 (*p* < 0.001). USV numbers in phase 1 and phase 3 were not significantly different. Analysis of behavioral parameters revealed no significant phase effects on durations of anogenital sniffing [*F*_(1, 9)_ = 0.65, NS], body sniffing [*F*_(1, 9)_ = 1.26, NS], follow [*F*_(1, 9)_ = 0.73, NS], and total social investigation [*F*_(1, 9)_ = 1.32, NS]. BTBR displayed higher levels of nose-to-nose sniffing in phase 3 than in phase 1 [*F*_(1, 9)_ = 5.97, *p* < 0.05].

### Call repertoires in the presence and absence of the female

Changes in call repertoires were detected when the female was removed from the arena and after the female was returned to the arena. As described in the figure legends, only individuals that emitted calls were used for the call category analyses.

**Figure 4:**
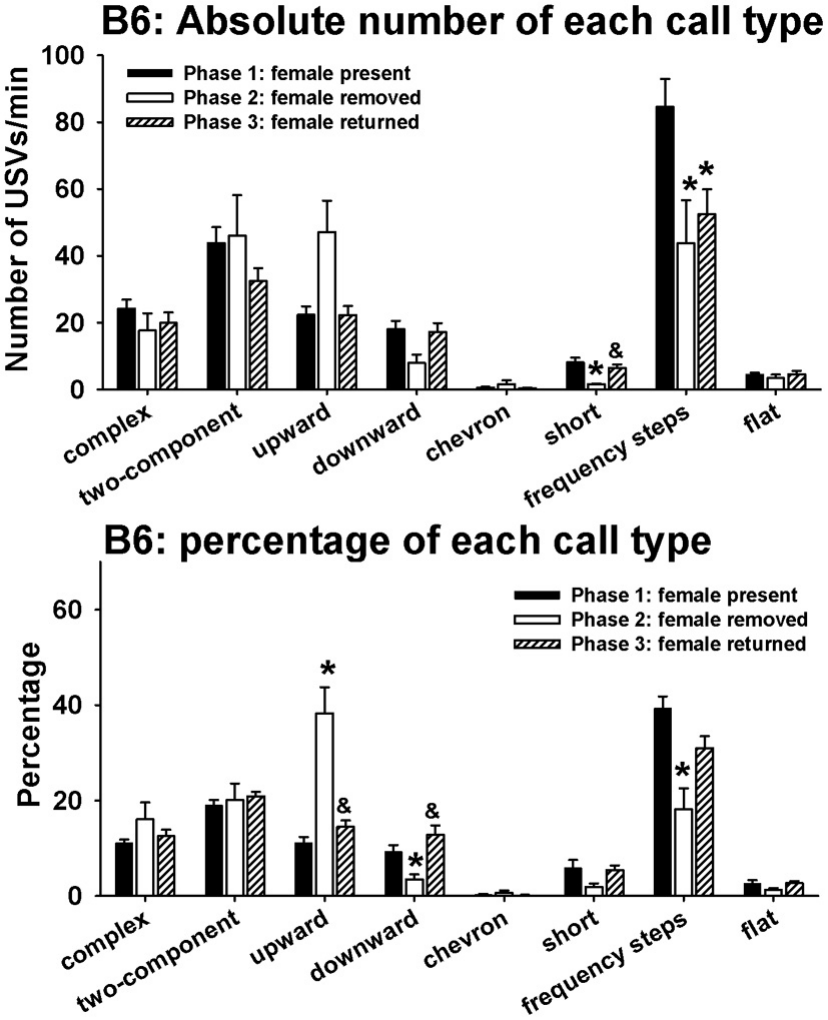
**Call repertoires in B6 males in phases 1, 2, and 3**. USVs were detected from 12 out of 12 B6 in phase 1, 4 out of 12 B6 in phase 2, and 10 out of 10 B6 in phase 3. Only animals that emitted USVs were included in call category analysis. Analysis of absolute values of USV numbers indicated significant phase effects on short and frequency steps. Fewer short and frequency steps were detected in phase 2 than in phase 1. More short were detected in phase 3 than in phase 2. Analysis of percentages of call types indicated significant phase effects on upward, downward, and frequency steps. Compared to phase 1, the percentage of upward was higher and the percentages of downward and frequency steps were lower in phase 2. These changes were reverted in phase 3, when the female returned. ^*^p < 0.05 vs. phase 1; ^&^*p* < 0.05 vs. phase 2.

Figure 4 illustrates call repertoires in B6 males in phases 1, 2, and 3. Analysis of absolute number of USVs in each call category indicated significant phase effects on short [*H*_(2)_ = 9.52, *p* < 0.01] and frequency steps [*H*_(2)_ = 9.44, *p* < 0.01]. Fewer short and frequency steps were detected in phase 2 than in phase 1 (*p* < 0.05 for each comparison). More short were detected in phase 3 than in phase 2 (*p* < 0.05). No significant phase effects were found for complex [*H*_(2)_ = 5.39, NS], two-component [*H*_(2)_ = 1.96, NS], upward [*H*_(2)_ = 5.13, NS], downward [*H*_(2)_ = 5.73, NS], chevron [*H*_(2)_ = 0.41, NS], and flat [*H*_(2)_ = 1.31, NS]. Analysis of percentage of each call category indicated significant phase effects on upward [*H*_(2)_ = 24.24, *p* < 0.001], downward [*H*_(2)_ = 13.21, *p* < 0.01], and frequency steps [*H*_(2)_ = 13.04, *p* < 0.01]. From phase 1 to phase 2, the percentage of upward increased whereas the percentages of downward and frequency steps decreased (*p* < 0.05 for each comparison). From phase 2 to phase 3, the percentage of upward decreased and the percentage of downward increased (*p* < 0.01). There was a trend for the percentage of frequency steps to increase from phase 2 to phase 3 (0.05 < *p* < 0.10, NS). No significant phase effects were found for complex [*H*_(2)_ = 0.79, NS], two-component [*H*_(2)_ = 1.91, NS], chevron [*H*_(2)_ = 0.25, NS], short [*H*_(2)_= 4.58, NS], and flat [*H*_(2)_ = 2.87, NS].

**Figure 5:**
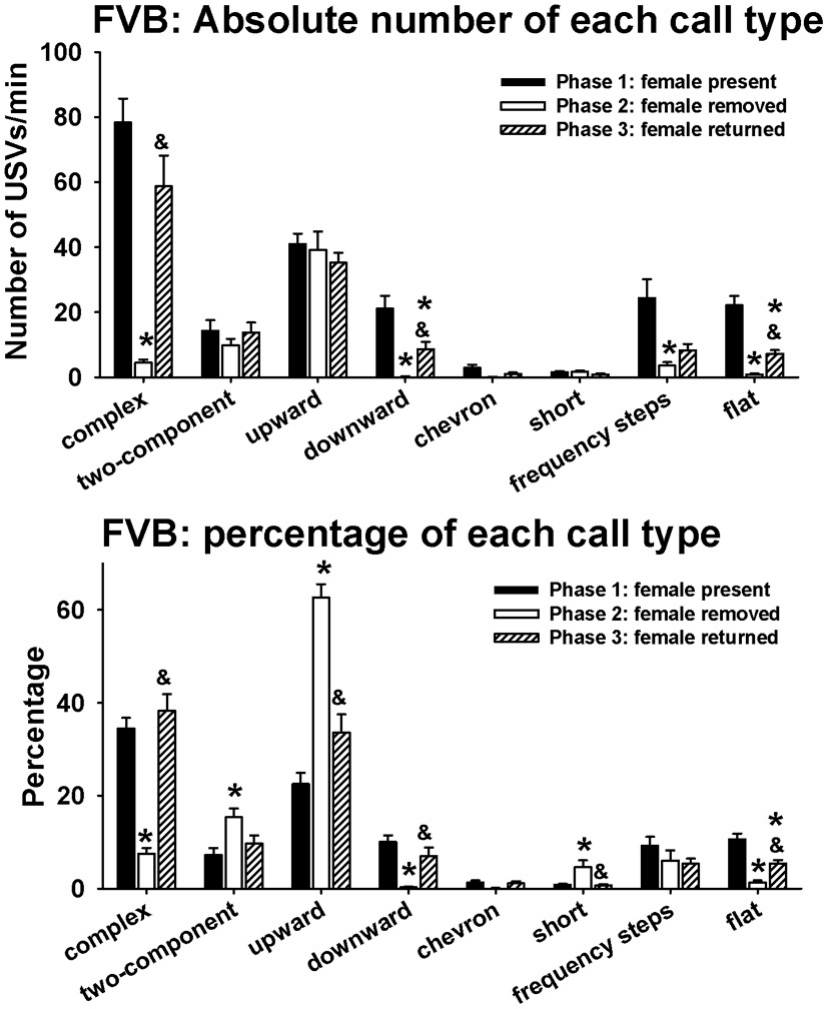
**Call repertoires in FVB males in phases 1, 2, and 3**. USVs were detected from 12 out of 12 FVB in phase 1, 12 out of 12 FVB in phase 2, and 10 out of 10 FVB in phase 3. Only animals that emitted USVs were included in call category analysis. Analysis of absolute values of USV numbers indicated significant phase effects on complex, downward, chevron, frequency steps, and flat. Numbers of complex, downward, frequency steps, and flat were lower in phase 2 than on phase 1. These changes were reverted in phase 3, when the female returned. Analysis of percentages of call types indicated significant phase effects on complex, two-component, upward, downward, short, and flat. Percentages of complex, downward, and flat were lower in phase 2 than in phase 1, and the percentages of two-component, upward, and short were higher in phase 2 than in phase 1. These changes were reverted in phase 3, when the female returned. ^*^p < 0.05 vs. phase 1; ^&^*p* < 0.05 vs. phase 2.

Figure 5 illustrates call repertoires in FVB males in phases 1, 2, and 3. Analysis of absolute number of USVs in each call category indicated significant phase effects on complex [*H*_(2)_ = 58.04, *p* < 0.001], downward [*H*_(2)_ = 57.71, *p* < 0.001], chevron [*H*_(2)_ = 10.04, *p* < 0.01], frequency steps [*H*_(2)_ = 16.50, *p* < 0.001], and flat [*H*_(2)_ = 61.96, *p* < 0.001]. Fewer complex, downward, frequency steps, and flat were detected in phase 2 than in phase 1 (*p* < 0.01 for each comparison). More complex, downward, and flat were detected in phase 3 than in phase 2 (*p* < 0.01 for each comparison). Downward and flat were lower in phase 3 than in phase 1 (*p* < 0.05 for each comparison). No pairwise differences were found for chevron. No significant phase effects were found for two-component [*H*_(2)_ = 2.24, NS], upward [*H*_(2)_ = 1.25, NS], and short [*H*_(2)_= 4.94, NS]. Analysis of percentage of each call category indicated significant phase effects on complex [*H*_(2)_ = 54.91, *p* < 0.001], two-component [*H*_(2)_ = 13.59, *p* < 0.001], upward [*H*_(2)_ = 46.93, *p* < 0.01], downward [*H*_(2)_ = 51.05, *p* < 0.001], short [*H*_(2)_ = 7.46, *p* < 0.05], and flat [*H*_(2)_ = 49.43, *p* < 0.001]. Lower percentages of complex, downward, and flat, and higher percentages of two-component, upward, and short were detected in phase 2 than in phase 1 (*p* < 0.01 for each comparison). Higher percentages of complex, downward, and flat (*p* < 0.01 for each comparison), and lower percentages of upward and short (*p* < 0.05 for each comparison) were detected in phase 3 than in phase 2. No significant phase effects were found for frequency steps [*H*_(2)_ = 5.58, NS].

**Figure 6:**
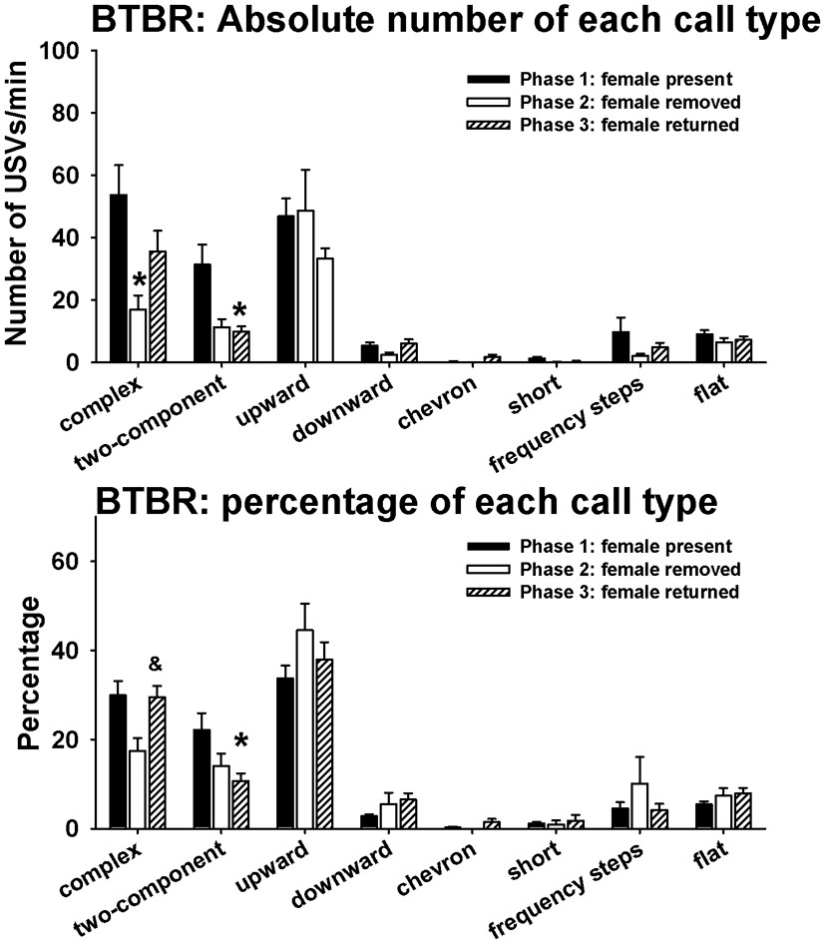
**Call repertoires in BTBR males in phases 1, 2, and 3**. USVs were detected from 9 out of 11 BTBR in phase 1, 4 out of 11 BTBR in phase 2, and 9 out of 11 BTBR in phase 3. Only animals that emitted USVs were included in call category analysis. Analysis of absolute call numbers indicated significant phase effects on complex and two-component. Number of complex was lower in phase 2 than in phase 1. Number of two-component was lower in phase 3 than in phase 1. Analysis of percentages of call types indicated significant phase effects on complex and two-component. A trend was found for the percentage of complex to decrease in phase 2. The percentage of complex was higher in phase 3 than in phase 2. The percentage of two-component was lower in phase 3 than in phase 1. ^*^p < 0.05 vs. phase 1; ^&^*p* < 0.05 vs. phase 2.

Figure 6 illustrates call repertoires in BTBR males in phases 1, 2, and 3. Analysis of absolute number of USVs in each call category indicated significant phase effects on complex [*H*_(2)_ = 6.52, *p* < 0.05] and two-component [*H*_(2)_ = 8.92, *p* < 0.01]. Fewer complex calls were detected in phase 2 than in phase 1 (*p* < 0.05). Fewer two-component calls were detected in phase 3 than in phase 1 (*p* < 0.01). No significant phase effects were found on upward [*H*_(2)_ = 2.67, NS], downward [*H*_(2)_ = 2.58, NS], chevron [*H*_(2)_ = 2.62, NS], short [*H*_(2)_ = 7.52, NS], frequency steps [*H*_(2)_ = 1.72, NS], and flat [*H*_(2)_ = 0.68, NS]. Analysis of percentage of each call type indicated significant phase effects on complex [*H*_(2)_ = 6.81, *p* < 0.05] and two-component [*H*_(2)_ = 8.99, *p* < 0.01]. A trend was found for the percentage of complex to be lower in phase 2 than in phase 1 (0.05 < p < 0.10). The percentage of complex was higher in phase 3 than in phase 2 (*p* < 0.05). The percentage of two-component was lower in phase 3 than in phase 1 (*p* < 0.01). No significant phase effects were found on upward [*H*_(2)_ = 2.35, NS], downward [*H*_(2)_ = 4.62, NS], chevron [*H*_(2)_ = 2.52, NS], short [*H*_(2)_ = 5.20, NS], frequency steps [*H*_(2)_ = 0.61, NS], and flat [*H*_(2)_ = 1.70, NS].

### Strain differences in USVs in the presence and absence of the female

Significant strain differences in number of USVs were found in the first 3 min of phase 1 [B6: 693.0 ± 50.8; FVB: 726.4 ± 47.5; BTBR: 397.5 ± 93.2, *F*_(2, 32)_ = 7.46, *p* < 0.01], phase 2 [B6: 154.8.0 ± 86.4; FVB: 196.2 ± 33.0; BTBR: 87.9 ± 50.7, *H*_(2)_ = 7.40, *p* < 0.05], and phase 3 (B6: 455.1 ± 66.7; FVB: 389.6 ± 50.0; BTBR: 236.8 ± 60.2, *F*_(2, 32)_ = 3.63, *p* < 0.05]. In phase 1, fewer calls were detected in pairs of BTBR males and stimulus females than in pairs of B6 males and stimulus females and pairs of FVB males and stimulus females (*p* < 0.01 for each comparison). The difference between B6 and FVB was not significant. In phase 2, a trend was detected for BTBR to emit fewer USVs than FVB (*p* < 0.062). In phase 3, BTBR emitted fewer USVs than B6 (*p* < 0.05). The difference between B6 and FVB was not significant.

Strain differences in call categories in phases 1, 2, and 3 were expressed as absolute values and as percentage values (Table 1). Analysis of absolute numbers of USVs in each call category indicated significant strain differences in phase 1, on numbers of complex [*H*_(2)_ = 12.90, *p* < 0.01], two-component [*H*_(2)_ = 11.43, *p* < 0.01], upward [*H*_(2)_ = 9.30, *p* < 0.01], downward [*H*_(2)_ = 11.33, *p* < 0.01], short [*H*_(2)_ = 14.70, *p* < 0.01], frequency steps [*H*_(2)_ = 17.92, *p* < 0.001], and flat [*H*_(2)_ = 18.56, *p* < 0.001]. No significant strain differences were found for chevron [*H*_(2)_ = 1.88, NS]. Compared to B6, FVB emitted more complex (*p* < 0.001), upward (*p* < 0.05), flat (*p* < 0.001), and fewer two-component (*p* < 0.01), short (*p* < 0.01), and frequency steps (*p* < 0.01). Compared to B6, BTBR emitted more upward (*p* < 0.05), and fewer downward (*p* < 0.05), short (*p* < 0.01), and frequency steps (*p* < 0.01). In phase 2, significant strain differences were found in numbers of downward [*H*_(2)_ = 10.23, *p* < 0.01] and short [*H*_(2)_ = 7.43, *p* < 0.05]. Compared to B6, FVB and BTBR emitted fewer downward (*p* < 0.05 for each comparison). BTBR emitted fewer short than FVB (*p* < 0.05). No significant strain differences were detected in numbers of complex [*H*_(2)_ = 4.63, NS], two-component [*H*_(2)_ = 2.30, NS], upward [*H*_(2)_ = 0.002, NS], chevron [*H*_(2)_ = 1.14, NS], frequency steps [*H*_(2)_ = 2.62, NS], and flat [*H*_(2)_ = 4.23, NS]. In phase 3, strain differences were somewhat similar to those seen in phase 1. Significant strain differences were detected in numbers of complex [*H*_(2)_ = 8.35, *p* < 0.05], two-component [*H*_(2)_ = 13.00, *p* < 0.01], downward [*H*_(2)_ = 7.30, *p* < 0.05], short [*H*_(2)_ = 15.94, *p* < 0.01], frequency steps [*H*_(2)_ = 14.74, *p* < 0.01]. Compared to B6, FVB emitted more complex (*p* < 0.01), and fewer two-component (*p* < 0.01), short (*p* < 0.01), and frequency steps (*p* < 0.05). Compared to B6, BTBR emitted fewer two-component (*p* < 0.01), downward (*p* < 0.05), short (*p* < 0.01), and frequency steps (*p* < 0.01). No significant strain effects were found on upward [*H*_(2)_ = 3.15, NS], chevron [*H*_(2)_ = 1.36, NS], and flat [*H*_(2)_ = 2.39, NS].

**Table 1:** **Strain differences in call categories during the first interaction with the female (phase 1), absence of the female (phase 2), and second interaction with the female (phase 3)**.

**Call category**	**B6**	**FVB**	**BTBR**	**Strain effect (*p*-values)**
**PHASE 1: TOTAL CALLS IN MINUTES 1, 3, AND 5**				
Complex	72.5 ± 7.9	235.0 ± 29.1^*^	161.0 ± 46.4	*p* < 0.01
Two-component	131.4 ± 19.9	42.8 ± 14.5^*^	93.9 ± 25.6	*p* < 0.01
Upward	67.0 ± 9.5	122.8 ± 14.9^*^	140.4 ± 22.8^*^	*p* < 0.01
Downward	54.0 ± 11.4	63.3 ± 12.2	15.9 ± 4.3^*^	*p* < 0.01
Chevron	1.7 ± 0.76	8.6 ± 3.4	0.78 ± 0.36	NS
Short	24.1 ± 7.2	4.7 ± 1.0^*^	3.9 ± 1.6^*^	*p* < 0.01
Frequency steps	253.7 ± 31.7	73.1 ± 24.5^*^	29.0 ± 15.5^*^	*p* < 0.001
Flat	13.2 ± 1.7	66.4 ± 11.9^*^	26.9 ± 6.1	*p* < 0.001
**PHASE 2: TOTAL CALLS IN MINUTES 1, 2, AND 3**				
Complex	48.5 ± 23.0	12.9 ± 2.8	46.5 ± 24.8	NS
Two-component	126.5 ± 58.8	29.0 ± 7.6	31.0 ± 11.1	NS
Upward	129.5 ± 45.1	115.3 ± 21.5	133.8 ± 68.0	NS
Downward	21.8 ± 10.9	0.58 ± 0.29^*^	6.8 ± 1.8^*^	*p* < 0.01
Chevron	4.3 ± 4.3	0.17 ± 0.11	0.0 ± 0.0	NS
Short	4.3 ± 1.0	5.2 ± 1.7	0.25 ± 0.25^#^	*p* < 0.05
Frequency steps	120.3 ± 55.1	10.5 ± 4.3	5.75 ± 2.3	NS
Flat	9.3 ± 5.0	2.5 ± 1.0	17.8 ± 6.5	NS
**PHASE 3: TOTAL CALLS IN MINUTES 1, 2, AND 3**				
Complex	59.1 ± 13.1	176.2 ± 41.7^*^	102.8 ± 28.3	*p* < 0.05
Two-component	99.6 ± 14.7	41.3 ± 14.4^*^	28.4 ± 7.4^*^	*p* < 0.01
Upward	68.4 ± 12.7	105.7 ± 10.6	96.0 ± 17.5	NS
Downward	52.4 ± 12.0	25.9 ± 10.9	17.7 ± 5.8^*^	*p* < 0.05
Chevron	1.1 ± 0.61	3.2 ± 1.4	5.0 ± 4.0	NS
Short	19.3 ± 4.75	2.6 ± 1.1^*^	1.1 ± 0.31^*^	*p* < 0.01
Frequency steps	157.3 ± 29.1	24.9 ± 8.2^*^	14.0 ± 6.8^*^	*p* < 0.01
Flat	13.8 ± 3.1	21.7 ± 4.5	24.9 ± 6.3	NS
**PHASE 1: PERCENTAGE VALUES OF TOTAL CALLS IN MINUTES 1, 3, AND 5**				
Complex	12.0 ± 0.90	37.3 ± 3.3^*^	30.3 ± 4.7^*^	*p* < 0.001
Two-component	21.0 ± 1.5	6.9 ± 2.3^*^	19.5 ± 3.1^#^	*p* < 0.01
Upward	11.6 ± 1.6	20.9 ± 2.6^*^	36.5 ± 5.1^*^	*p* < 0.01
Downward	9.4 ± 2.4	11.3 ± 2.2	2.9 ± 0.68^*^	*p* < 0.01
Chevron	0.28 ± 0.11	1.4 ± 0.60	0.31 ± 0.18	NS
Short	4.8 ± 1.7	0.83 ± 0.19^*^	1.4 ± 0.48	*p* < 0.05
Frequency steps	40.5 ± 3.5	10.0 ± 2.8^*^	5.6 ± 2.6^*^	*p* < 0.001
Flat	2.3 ± 0.28	11.4 ± 2.1^*^	5.9 ± 0.57^*^	*p* < 0.001
**PHASE 2: PERCENTAGE VALUES OF TOTAL CALLS IN MINUTES 1, 2, AND 3**				
Complex	15.2 ± 5.6	9.3 ± 2.3	16.3 ± 3.9	NS
Two-component	21.1 ± 5.6	14.8 ± 2.2	15.3 ± 2.7	NS
Upward	37.3 ± 9.1	63.0 ± 6.5	41.7 ± 10.9	NS
Downward	3.7 ± 1.6	0.24 ± 0.12	8.2 ± 5.1^#^	*p* < 0.01
Chevron	0.47 ± 0.47	0.12 ± 0.09	0.0 ± 0.0	NS
Short	1.9 ± 1.0	6.8 ± 2.9	1.9 ± 1.9	NS
Frequency steps	18.9 ± 6.7	6.4 ± 1.8	10.1 ± 7.0	NS
Flat	1.3 ± 0.59	1.0 ± 0.43	6.5 ± 2.5	NS
**PHASE 3: PERCENTAGE VALUES OF TOTAL CALLS IN MINUTES 1, 2, AND 3**				
Complex	14.3 ± 2.6	40.8 ± 5.4^*^	41.0 ± 8.2^*^	*p* < 0.01
Two-component	24.9 ± 3.5	9.8 ± 2.6^*^	10.3 ± 3.0^*^	*p* < 0.01
Upward	16.5 ± 3.2	30.0 ± 4.6^*^	37.5 ± 3.2^*^	*p* < 0.01
Downward	14.2 ± 3.6	7.0 ± 3.0	10.2 ± 5.1	NS
Chevron	0.19 ± 0.10	1.2 ±.54	1.5 ± 1.3	NS
Short	5.5 ± 1.5	0.64 ± 0.22^*^	5.9 ± 5.5^*^	*p* < 0.01
Frequency steps	32.7 ± 4.4	5.7 ± 1.4^*^	3.6 ± 1.5^*^	*p* < 0.01
Flat	3.2 ± 0.51	5.7 ± 1.0	7.8 ± 1.8	NS

*Since phase 1 was 5 min. long, whereas phases 2 and 3 were 3 min. long, strain differences were analyzed by comparing total calls of each category in minutes 1, 3, and 5 of phase 1, total calls of each category in the 3 min. of phase 2, and total calls of each category in the 3 min of phase 3 among the three strains*.

*Data are mean ± S.E.M*.

* *p < 0.05 vs. B6*.

# *p < 0.05 vs. FVB. NS, not significant*.

Analysis of percentage data indicated significant strain differences in phase 1, on percentages of complex [*H*_(2)_ = 22.68, *p* < 0.001], two-component [*H*_(2)_ = 13.50, *p* < 0.01], upward [*H*_(2)_ = 17.78, *p* < 0.01], downward [*H*_(2)_ = 10.68, *p* < 0.01], short [*H*_(2)_ = 8.61, *p* < 0.05], frequency steps [*H*_(2)_ = 20.97, *p* < 0.001], and flat [*H*_(2)_ = 23.03, *p* < 0.001]. No significant strain differences were found on chevron [*H*_(2)_ = 1.61, NS]. Compared to B6, FVB had higher percentages of complex (*p* < 0.001), upward (*p* < 0.01), flat (*p* < 0.001), and lower percentages of two-component (*p* < 0.01), short (*p* < 0.05), and frequency steps (*p* < 0.001). Compared to B6, BTBR had higher percentages of complex (*p* < 0.01), upward (*p* < 0.01), flat (*p* < 0.01), and lower percentages of downward (*p* < 0.05) and frequency steps (*p* < 0.001). Compared to FVB, BTBR had higher percentages of two-component (*p* < 0.01). In phase 2, significant strain differences were found in percentages of downward [*H*_(2)_ = 10.86, *p* < 0.01]. BTBR had a higher percentage of downward than FVB (*p* < 0.05). No significant strain differences were found in complex [*H*_(2)_ = 3.11, NS], two-components [*H*_(2)_ = 2.30, NS], upward [*H*_(2)_ = 5.0, NS], chevron [*H*_(2)_ = 1.13, NS], short [*H*_(2)_= 3.28, NS], frequency steps [*H*_(2)_ = 1.18, NS], and flat [*H*_(2)_ = 4.13, NS]. In phase 3, strain differences were similar to those seen in phase 1. Significant strain differences were found in percentages of complex [*H*_(2)_ = 15.03, *p* < 0.01], two-component [*H*_(2)_ = 11.23, *p* < 0.01], upward [*H*_(2)_ = 12.82, *p* < 0.01], short [*H*_(2)_ = 10.37, *p* < 0.01], frequency steps [*H*_(2)_ = 17.71, *p* < 0.01]. No significant strain differences were found in downward [*H*_(2)_ = 5.19, NS], chevron [*H*_(2)_ = 1.69, NS], and flat [*H*_(2)_ = 5.04, NS]. Compared to B6, FVB had higher percentages of complex (*p* < 0.01), upward (*p* < 0.05), and lower percentages of two-component (*p* < 0.01), short (*p* < 0.05), and frequency steps (*p* < 0.01). Compared to B6, BTBR had higher percentages of complex (*p* < 0.01), upward (*p* < 0.01), and lower percentages of two-component (*p* < 0.05), short (*p* < 0.05), and frequency steps (*p* < 0.01).

### Strain differences in social behaviors

Male subject mice of all three strains displayed the expected social interactions with the estrus female. As predicted from previous reports (Silverman et al., 2010a; Scattoni et al., 2011), B6 and FVB engaged in more interactions than BTBR (Table 2). In phase 1, significant strain differences were found in durations of nose-to-nose sniffing [*F*_(2, 31)_ = 5.42, *p* < 0.01], anogenital sniffing [*F*_(2, 31)_ = 30.11, *p* < 0.001], body sniffing [*F*_(2, 31)_ = 6.13, *p* < 0.01], and follow [*F*_(2, 31)_ = 19.12, *p* < 0.001]. Total social investigation time also differed significantly across strains [*F*_(2, 31)_ = 46.73, *p* < 0.001]. As compared to B6, BTBR exhibited shorter durations of total social investigation (*p* < 0.01) and trends to display shorter durations of nose-to-nose sniffing (*p* < 0.059), anogenital sniffing (*p* < 0.058), and follow (*p* < 0.058). As compared to FVB, BTBR had shorter durations of all social behaviors (*p* < 0.01 for each comparison). As compared to B6, FVB exhibited significantly longer durations of anogenital sniffing (*p* < 0.01), follow (*p* < 0.01), and total social investigation (*p* < 0.01). Analysis of numbers of behavioral parameters revealed similar results. Significant strain differences were found in numbers of nose-to-nose sniffing [*F*_(2, 31)_ = 4.50, *p* < 0.05], anogenital sniffing [*F*_(2, 31)_ = 4.47, *p* < 0.05], and follow [*F*_(2, 31)_ = 7.23, *p* < 0.01]. Number of total social investigation also differed significantly across strains [*F*_(2, 31)_ = 7.12, *p* < 0.01]. No strain differences were found for bouts of arena exploration [*F*_(2, 31)_ = 2.80, NS]. As compared to B6, BTBR had fewer counts of nose-to-nose sniffing (*p* < 0.05) and total social investigation (*p* < 0.05). As compared to FVB, BTBR had fewer counts of follow (*p* < 0.01), anogenital sniffing (*p* < 0.05) and total social investigation (*p* < 0.01). No significant differences were found between B6 and FVB on counts of behaviors.

**Table 2:** **Strain differences in male social behaviors during the first interaction with the female (phase 1) and second interaction with the female (phase 3)**.

**PHASE 1 (5 min)**				
**Behavior (s/5min)**	**B6**	**FVB**	**BTBR**	**Strain effect (*p*-values)**
Nose-to-nose sniffing	14.6 ± 2.4	20.2 ± 3.6	6.6 ± 2.2^#^	*p* < 0.01
Anogenital sniffing	37.2 ± 6.2	82.2 ± 5.9^*^	21.1 ± 4.8^#^	*p* < 0.001
Body sniffing	7.8 ± 2.2	18.4 ± 4.6	3.0 ± 1.1^#^	*p* < 0.01
Follow	22.4 ± 4.7	51.8 ± 6.3^*^	8.3 ± 3.0^#^	*p* < 0.001
Total social investigation	82.0 ± 9.5	172.6 ± 10.3^*^	39.1 ± 9.8^*^^#^	*p* < 0.001
**Behavior (counts/5min)**	**B6**	**FVB**	**BTBR**	**Strain effect (*p*-values)**
Nose-to-nose sniffing	9.4 ± 0.61	7.3 ± 1.2	5.1 ± 1.5^*^	*p* < 0.05
Anogenital sniffing	10.5 ± 1.54	14.0 ± 0.97	8.5 ± 1.2^#^	*p* < 0.05
Body sniffing	3.42 ± 0.92	5.9 ± 1.2	2.5 ± 0.89	NS
Follow	9.5 ± 1.9	12.8 ± 1.2	4.5 ± 1.2^#^	*p* < 0.01
Total social investigation	32.8 ± 3.5	40.0 ± 3.4	20.6 ± 4.0^*^^#^	*p* < 0.01
Arena exploration	7.4 ± 1.0	7.7 ± 0.89	11.2 ± 1.81	NS
**PHASE 3 (3 min)**				
**Behavior (s/3 min)**	**B6**	**FVB**	**BTBR**	**Strain effect (*p*-values)**
Nose-to-nose sniffing	16.2 ± 4.3	29.0 ± 4.8	11.6 ± 3.8^#^	*p* < 0.05
Anogenital sniffing	25.4 ± 4.3	33.6 ± 4.6	9.9 ± 2.6^*^^#^	*p* < 0.001
Body sniffing	5.7 ± 1.3	21.6 ± 5.1^*^	3.0 ± 0.93^#^	*p* < 0.001
Follow	10.1 ± 3.4	25.3 ± 2.5^*^	7.2 ± 2.2^#^	*p* < 0.001
Total social investigation	57.5 ± 7.4	109.5 ± 7.3^*^	31.7 ± 6.3^*^^#^	*p* < 0.001
**Behavior (counts/3 min)**	**B6**	**FVB**	**BTBR**	**Strain effect (*p*-values)**
Nose-to-nose sniffing	7.4 ± 0.80	8.6 ± 0.69	5.2 ± 1.2^#^	*p* < 0.05
Anogenital sniffing	7.5 ± 1.0	7.3 ± 0.83	4.5 ± 0.73	*p* < 0.05
Body sniffing	3.2 ± 0.52	4.5 ± 0.91	2.4 ± 0.58	NS
Follow	3.3 ± 0.86	6.4 ± 0.65^*^	3.7 ± 1.0	*p* < 0.05
Total social investigation	21.4 ± 1.8	26.8 ± 1.2	15.8 ± 2.4^#^	*p* < 0.01
Arena exploration	3.8 ± 1.0	3.9 ± 0.71	4.9 ± 1.0	NS

Data are mean ± S.E.M.,

* p < 0.05 vs. B6.,

# *p < 0.05 vs. FVB. NS, not significant*.

In phase 3, significant strain differences were found in durations of nose-to-nose sniffing [*F*_(2, 29)_ = 4.02, *p* < 0.05], anogenital sniffing [*F*_(2, 29)_ = 8.63, *p* < 0.001], body sniffing [*F*_(2, 29)_ = 11.17, *p* < 0.001], and follow [*F*_(2, 29)_ = 10.92, *p* < 0.001]. Total social investigation time also differed significantly across strains [*F*_(2, 29)_ = 29.23, *p* < 0.001]. As compared to B6, BTBR exhibited shorter durations of anogenital sniffing (*p* < 0.05) and total social investigation (*p* < 0.05). As compared to FVB, BTBR had shorter durations of all social behaviors (*p* < 0.05 for each comparison). As compared to B6, FVB exhibited significantly higher levels of body sniffing (*p* < 0.01), follow (*p* < 0.01), and total social investigation (*p* < 0.01). Analysis of numbers of behavioral parameters revealed similar results. Significant strain differences were found in numbers of nose-to-nose sniffing [*F*_(2, 29)_ = 3.27, *p* < 0.05], anogenital sniffing [*F*_(2, 29)_ = 3.39, *p* < 0.05], and follow [*F*_(2, 29)_ = 3.67, *p* < 0.05]. Number of total social investigation also differed significantly across strains [*F*_(2, 29)_ = 8.26, *p* < 0.01]. No strain differences were found for bouts of arena exploration [*F*_(2, 29)_ = 0.44, NS]. As compared to B6, BTBR exhibited a trend toward fewer counts of anogenital sniffing (*p* < 0.07, NS). As compared to B6, FVB exhibited more counts of follow (*p* < 0.05). As compared to FVB, BTBR exhibited fewer counts of nose-to-nose sniffing (*p* < 0.05) and total social investigation (*p* < 0.01).

Analysis of behaviors of female stimulus mice paired with three strains of males revealed minimum effects of the male strain (Table 3). In phase 1, male strain had no significant effects on nose-to-nose sniffing [number: *F*_(2, 31)_ = 0.34, NS; duration: *F*_(2, 31)_ = 0.46, NS], body sniffing (number *F*_(2, 31)_ = 1.30, NS; duration: *F*_(2, 31)_ = 1.85, NS], follow [number: *F*_(2, 31)_ = 1.04, NS; duration: *F*_(2, 31)_ = 1.11, NS], total social investigation [number: *F*_(2, 31)_ = 0.21, NS; duration: *F*_(2, 31)_ = 0.61, NS], and bouts of arena exploration [*F*_(2, 31)_ = 1.02, NS]. Male strain had a significant effect on anogenital sniffing [number: *F*_(2, 31)_ = 4.82, *p* < 0.05; duration: *F*_(2, 31)_ = 3.24, *p* < 0.05]. As compared to females paired with B6 males, females paired with FVB males exhibited lower levels of anogenital sniffing (*p* < 0.05). In phase 3, male strain had no significant effects on nose-to-nose sniffing [number: *F*_(2, 29)_ = 1.22, NS; duration: *F*_(2, 29)_ = 2.42, NS], body sniffing [number *F*_(2, 29)_ = 2.09, NS; duration: *F*_(2, 29)_ = 1.54, NS], anogenital sniffing [number: *F*_(2, 29)_ = 1.54, NS; *F*_(2, 29)_ = 1.02, NS], follow [number: *F*_(2, 29)_ = 0.54, NS; duration: *F*_(2, 29)_ = 0.87, NS], total social investigation [number: *F*_(2, 29)_ = 0.43, NS; duration: *F*_(2, 29)_ = 0.08, NS]. Male strain had a significant effect on bouts of arena exploration [*F*_(2, 29)_ = 3.69, *p* < 0.05]. As compared to females paired with B6 males, females paired with FVB males and females paired with BTBR males exhibited more bouts of arena exploration (*p* < 0.05 for each comparison).

**Table 3:** **Behaviors of stimulus B6 females during phase 1 and phase 3**.

**PHASE 1 (5 min)**				
**Behavior (s/5 min)**	**Females paired with B6 males**	**Females paired with FVB males**	**Females paired with BTBR males**	**Effect of male strain (*p*-values)**
Nose-to-nose sniffing	2.2 ± 0.55	3.0 ± 0.51	3.6 ± 1.7	NS
Anogenital sniffing	0.84 ± 0.36	0 ± 0^*^	0.30 ± 0.13	*p* < 0.05
Body sniffing	0.14 ± 0.07	0.82 ± 0.37	0.59 ± 0.24	NS
Follow	0.22 ± 0.11	0.93 ± 0.50	0.78 ± 0.34	NS
Total social investigation	3.4 ± 0.57	4.7 ± 0.89	5.28 ± 1.93	NS
**Behavior (counts/5 min)**	**Females paired with B6 males**	**Females paired with FVB males**	**Females paired with BTBR males**	**Effect of male strain (*p*-values)**
Nose-to-nose sniffing	4.2 ± 0.55	3.8 ± 0.51	3.5 ± 0.72	NS
Anogenital sniffing	0.90 ± 0.28	0 ± 0^*^	0.50 ± 0.22	*p* < 0.05
Body sniffing	0.30 ± 0.15	0.80 ± 0.25	0.80 ± 0.33	NS
Follow	0.30 ± 0.15	0.40 ± 0.16	0.80 ± 0.39	NS
Total social investigation	5.7 ± 0.67	5.0 ± 0.65	5.6 ± 1.1	NS
Arena exploration	13.1 ± 1.1	15.5 ± 1.3	14.1 ± 1.2	NS
**PHASE 3 (3 min)**				
**Behavior (s/3 min)**	**Females paired with B6 males**	**Females paired with FVB males**	**Females paired with BTBR males**	**Effect of male strain (*p*-values)**
Nose-to-nose sniffing	1.8 ± 0.53	0.1.9 ± 0.51	0.73 ± 0.18	NS
Anogenital sniffing	0 ± 0	0.24 ± 0.13	0.22 ± 0.17	NS
Body sniffing	0 ± 0	0.11 ± 0.11	0.25 ± 0.14	NS
Follow	0.28 ± 0.19	0.31 ± 0.11	1.1 ± 0.80	NS
Total social investigation	2.1 ± 0.62	2.6 ± 0.64	2.3 ± 1.1	NS
**Behavior (counts/3 min)**	**Females paired with B6 males**	**Females paired with FVB males**	**Females paired with BTBR males**	**Effect of male strain (*p*-values)**
Nose-to-nose sniffing	3.8 ± 1.1	3.0 ± 0.52	1.8 ± 0.36	NS
Anogenital sniffing	0 ± 0	0.30 ± 0.15	0.20 ± 0.13	NS
Body sniffing	0 ± 0	0.20 ± 0.20	0.40 ± 0.22	NS
Follow	0.33 ± 0.17	0.20 ± 0.20	0.60 ± 0.40	NS
Total social investigation	4.1 ± 1.2	3.7 ± 0.52	3.0 ± 0.80	NS
Arena exploration	5.7 ± 0.53	8.4 ± 0.52^*^	7.9 ± 1.0^*^	*p* < 0.05

*Data are mean ± S.E.M*.

* *, p < 0.05 vs. B6. NS, not significant*.

## Discussion

Large numbers of complex USVs are detectable when adult male mice interact with receptive females (Holy and Guo, 2005; Portfors, 2007; Hammerschmidt et al., 2009, Fishcher and Hammerschmidt, 2011 Kikusui et al., 2011; Scattoni et al., 2011; Arriaga et al., 2012; Hammerschmidt et al., 2012b; Hanson and Hurley, 2012; Arriaga and Jarvis, 2013; Ey et al., 2013; Mahrt et al., 2013). The role of USVs in male-female social interaction was first investigated four decades ago by Whitney and colleagues (Whitney et al., 1973). Subsequent studies indicated that female mice preferred male USVs over pup USVs, artificial control sounds, and silence (Hammerschmidt et al., 2009; Shepard and Liu, 2011), preferred vocalizing males over devocalized males (Pomerantz et al., 1983), and preferred USVs from non-kin males over USVs from kin males (Musolf et al., 2010). In a pioneering study, Holy and Guo (Holy and Guo, 2005) demonstrated the complexity of male calls during male-female interaction, and suggested that the male calls have characteristics of songs. Several other groups that used different categorizing criteria also reported complex call repertoires in inbred mouse strains (Kikusui et al., 2011; Scattoni et al., 2011) and transgenic mouse models (Wang et al., 2008; Ey et al., 2012; Hammerschmidt et al., 2012b; Roy et al., 2012; Srivastava et al., 2012; Mahrt et al., 2013). Large number of complex USVs are also detectable during same-sex interactions in adult mice (Scattoni et al., 2011; Ey et al., 2012; Hammerschmidt et al., 2012a; Ey et al., 2013). Little evidence exists on whether sudden changes in social cues would result in changes in call number and call repertoire (Hanson and Hurley, 2012). In the current study we investigated quantitative and qualitative differences in USVs when the male interacts with a female and after the removal of the female in three inbred strains of mice.

One major finding of the present study is that three inbred strains with high and low sociability scores vocalized after the female was removed, indicating that post-interaction calls may be detected in many other strains and lines of mice and may be useful in studying vocal communication in mice. All three strains of naïve males emitted fewer calls in the absence of the female than in the presence of the female. All FVB males and one third each of B6 and BTBR males emitted calls in phase 2, indicating strain differences in response to the removal of salient social cues. To our knowledge, only one previous study reported USVs emitted by male mice after the removal of the female (Hanson and Hurley, 2012). Our results suggest that removing salient social cues might be a unique approach to elicit USVs in mice. It is also possible that the males vocalized not to the removal of the female *per se*, but to the sudden change in the environment, as novelty was shown to be effective in eliciting USVs in mice (Chabout et al., 2012).

The second finding of our study is that call repertoires changed after the female was removed, and reverted back when the female returned. These changes are particularly clear in the highly social B6 and FVB groups. Only one call type underwent changes in the low social BTBR group. In pairs of B6 males and stimulus females, large numbers of complex, two-component, upward, downward, and frequency steps were detected in phase 1. Fewer short, frequency steps, and trends toward more upward and fewer downward calls were detected when the female was removed. Changes in upward, downward, and short reverted back to phase 1 levels when the female returned. Analysis of percentage of each type of call across the three phases indicated an increase in upward, and decreases in downward and frequency steps when the female was removed. These changes reverted back to phase 1 levels when the female returned. In pairs of FVB males and stimulus females, large numbers of complex, upward, downward, frequency steps and flat were detected in phase 1. When the female was removed, the number of upward remained high while numbers complex, downward, frequency steps, and flat decreased markedly. Changes in numbers of complex and downward reverted back to phase 1 levels when the female was returned. Analysis of percentage of each type of call indicated significant decreases in complex, downward, flat and increases in two-component, upward, and short when the female was removed. All these changes reverted to phase 1 levels when the female returned. In pairs of BTBR males and stimulus females, a decrease in complex was detected when the female was removed. This change reverted to phase 1 level when the female returned. These findings provide evidence on which call types may be sensitive to the presence of the female. Removal of the female resulted in robust increases in upward and decreases in downward in B6 and FVB. Upward represented almost 40% of calls in B6 and more than 60% of calls in FVB after the female was removed. It is an intriguing possibility that upward calls may function to attract females. Playback experiments are needed to test the functional value of upward calls. Three call types in B6, six call types in FVB, and only one call type in BTBR changed across phases. It is conceivable that flexibility in vocal repertoires is related to the ability to sense changes in social situation or interests in social cues.

As we were preparing our manuscript, a similar study was published by Hanson and Hurley (Hanson and Hurley, 2012). Their paradigm focused on phases 1 and 2 only, i.e., a 5-min male-female interaction session and 5-min session after the female was removed. Interestingly, their data indicated that CBA/J males made more calls after the female was removed. In our study, B6, FVB, and BTBR males made fewer calls after the female was removed. Several important differences in environmental factors and procedural differences could have contributed to the discrepancy between the two studies. In the Hanson and Hurley study, males and females were all singly housed, and the males were vasectomized. Further, each male had multiple interactions with multiple females before being tested with a familiar female, the males were tested for multiple times, and the test was done in the male's home cage. In our study, males and females were group-housed, each sexually naïve and physically intact male was tested with an unfamiliar female only once, and the test was conducted in a clean neutral cage. It is conceivable that some of these factors, especially social isolation and prior experience with females, could affect how males respond vocally to the removal of the female. Another possibility is that CBA/J males react to the removal of the female differently from B6, FVB, and BTBR males. Categorical data from the Hanson and Hurley study indicated a robust increase in upward calls and decreases in flat and downward calls after the female was removed. Similar results were found in FVB and B6 males in our study, indicating that upward might be a call unique to the sudden absence of a social partner.

During the two sessions of male-female interactions, BTBR males exhibited lower levels of social sniffing and emitted fewer USVs than B6 males. These data are highly consistent with a previous study (Scattoni et al., 2011). It is notable that although B6 and BTBR differed significantly on total social investigation (sum of nose-to-nose sniffing, anogenital sniffing, body sniffing, and follow), they were not statistically different on each individual behavior. In accordance with their prolific breeding, it appears that BTBR mice are not grossly impaired in sexual approach behaviors. The finding that FVB males exhibited extremely high levels of social sniffing indicates that FVB could be used as second highly social control strain to corroborate findings in B6. The FVB/Ant strain has not been extensively studied. Future experiments need to test other phenotypes that are relevant to the interpretation of its social behaviors. Since behaviors of the females could influence behaviors of the males, we analyzed behaviors of all stimulus females. Results indicated that behaviors of the females were similar regardless the strain of the male. In phase 1, the only difference was that females paired with B6 males exhibited more anogenital sniffing than females paired with FVB males. In phase 3, male strain had no significant effects on social behaviors of the females.

Significant strain differences in call repertoires were found among B6, FVB, and BTBR. Consistent with a previous study (Scattoni et al., 2011), current results indicated that BTBR emitted fewer frequency steps and short than B6 when the female was present. It is interesting to note that FVB and BTBR differed on call numbers and behavioral scores but had similar call repertoires. It is possible that call number and sociability are positively correlated and that call repertoire is less indicative of sociability. We were not able test these hypothesis in the current study, because USVs and behaviors were recorded with separate devices and the recordings were not synchronized. This prohibited us from analyzing whether certain types of calls always co-occur with certain behaviors. We are piloting methods to overcome this major limitation in our future studies. Another potential caveat in the current study is that B6 females were used as stimulus mice for all three strains of male subjects. To test whether strain differences in call repertoires are independent of the strain of the female, subsequent experiments will test males paired with females of the same strain. The third caveat is that we didn't have direct evidence to prove that all calls were from the males. It is possible that the females emitted some calls in the novel testing environment, for novelty exposure was shown to induce USVs in mice (Chabout et al., 2012).

Mouse USVs have been studied for decades with equipment such as the Bat Detector. Technical advances with higher fidelity microphones and digital software now permit detailed categorical analysis. At present, different research groups carry out spectrographic analysis using very different criteria, both for how many categories of calls should be analyzed and exactly how to define each category (Holy and Guo, 2005; Portfors, 2007; Scattoni et al., 2008; Wang et al., 2008; Fischer and Hammerschmidt, 2011; Grimsley et al., 2011; Kikusui et al., 2011; Chabout et al., 2012; Ey et al., 2012; Hammerschmidt et al., 2012b; Hanson and Hurley, 2012; Nakagawa et al., 2012; Arriaga and Jarvis, 2013; Mahrt et al., 2013). In the current study, calls were categorized using criteria based on several previous publications (Scattoni et al., 2008, 2011). However, results obtained using our three phase test could be different in other labs that employ different categorizing criteria. Present findings indicate that males of commonly used inbred strains emit USVs when a partner female leaves the testing arena, confirming a previous report (Hanson and Hurley, 2012), and further reveal that USVs are reinstated at a high level when the female returns. In addition, we discovered that call categories differ significantly when the female is absent than when the female is present, suggesting that removing a salient social stimulus may be a unique approach to investigate functions of mouse USVs. Until a standard classification scheme is adapted by most investigators, it is impossible to make meaningful comparisons on categorical results reported by different groups.

### Conflict of interest statement

The authors declare that the research was conducted in the absence of any commercial or financial relationships that could be construed as a potential conflict of interest.
